# Frequency of intravenous-to-oral antibiotic switch in VA hospitalized patients with community-acquired pneumonia

**DOI:** 10.1017/ice.2025.10389

**Published:** 2026-04

**Authors:** Logan Daniels, Brett Heintz, Brian Lund, Bruce Alexander, Daniel Livorsi

**Affiliations:** 1 Department of Pharmacy, Iowa City Veterans Affairs Health Care System, Iowa City, IA, USA; 2 Iowa City Veterans Affairs Health Care System, Iowa City, IA, USA; 3 Center for Access & Delivery Research and Evaluation, Iowa City Veterans Affairs Health Care System, Iowa City, IA, USA; 4 Department of Epidemiology, University of Iowa College of Public Health, Iowa City, IA, USA; 5 Division of Infectious Diseases, University of Iowa Carver College of Medicine, Iowa City, IA, USA

## Abstract

**Objective:**

Professional guidelines recommend an early switch from intravenous (IV)-to-oral antibiotics for community-acquired pneumonia (CAP) to facilitate early discharge and prevent hospital-related complications. However, it is unknown how often these IV-to-oral switches occur in clinical practice.

**Design:**

We performed a retrospective cohort study across 124 acute-care Veterans Administration hospitals to measure the frequency of early switches.

**Patients:**

Patient-admissions during 2018–2023 who had CAP and were started on IV antibiotics upon admission.

**Methods:**

We measured the percentage of hospitalized patients with CAP who had an early switch from IV-to-oral antibiotics, i.e., within 72 hours of admission. In addition, we calculated an observed-to-expected ratio for early switches at each hospital and compared a composite outcome (mortality and/or hospital readmission within 30 days of discharge) at hospitals with switch rates that were higher and lower than expected.

**Results:**

Of 31,183 patient-admissions for CAP, 17,282 (55.4%) were switched to oral antibiotics by day three of therapy. Overall, 5,629 (18.1%) died and/or were re-admitted within 30 days. The O:E ratio for early antibiotic switches ranged from 0.78 among hospitals in the lowest quartile to 1.23 in the highest quartile. There was no difference in the composite outcome across quartiles.

**Conclusion:**

Early switches from IV-to-oral antibiotics for CAP occurred in half of eligible cases. The frequency of these switches varied widely across facilities. Outcomes among patients at hospitals with high switch rates were comparable to outcomes at hospitals with low rates, thereby supporting the safety of early switches. More concerted efforts to promote these switches are needed.

## Introduction

Community-acquired pneumonia (CAP) is a common reason for hospitalization and antibiotic use.^1,2^ Inpatient treatment frequently begins with intravenous (IV) antibiotics, as recommended by clinical practice guidelines.^3,4^ However, a large body of evidence, including from randomized controlled trials, has shown that early switching from IV-to-oral antibiotics is safe and effective for CAP. This approach is associated with shorter length of stay, fewer days on antibiotics, and similar clinical outcomes compared to longer durations of IV antibiotic therapy.^5–10^ Thus, professional guidelines recommend timely transition from IV-to-oral antibiotics as soon as the patient is clinically stable, defined by improved vital signs and ability to ingest oral medications.^3,4^

Despite the efficacy and safety of early IV-to-oral antibiotic switches for CAP, data on the uptake of this practice is limited. A 2010–2015 review of 642 United States (U.S.) hospitals found that switches from IV-to-oral antibiotics occurred infrequently and varied widely across facilities.^8^ This study evaluated data from before the Joint Commission’s 2017 mandate for all hospitals to have antimicrobial stewardship programs, so the frequency of switches may have since improved.

In this study, we sought to provide a more updated national assessment of whether patients hospitalized with CAP are undergoing early switches from IV-to-oral antibiotics. We performed our analysis within the Veterans Health Administration (VHA) system, the largest integrated healthcare system in the United States. All VA hospitals have been required to have an antimicrobial stewardship program since 2014.

## Methods

We performed a retrospective cohort study among all patients hospitalized with CAP within the VHA during 2018–2023. The Institutional Review Board (IRB) of the University of Iowa and the Iowa City Veterans Health Care System approved this study. Waiver for informed consent was granted by the IRB for this retrospective cohort.

### Data sources

We accessed national administrative data from all VA facilities through the VA Corporate Data Warehouse (CDW) via the VA Informatics and Computing Infrastructure. In addition, we used the VA Support Service Center to gather data on hospital complexity. VA hospitals are scored according to their patient population, clinical services (e.g., intensive care unit [ICU] and surgery services), and education and research. A score of 1a is the most complex while a score of 3 is the least complex.

### Inclusion criteria

We included patient-admissions discharged from an observation or inpatient bed between January 1, 2018, and December 31, 2023, from a VA hospital who also had a principal discharge diagnosis, as defined by ICD-10 codes, of pneumonia or a principal discharge diagnosis of sepsis or acute respiratory failure with a secondary discharge diagnosis of pneumonia.^8^ The positive predictive value of this electronic criteria, based on our manual review of 50 cases, was 98%.

We included all relevant hospitalizations for the same patient even if they were admitted more than once throughout the study period. To be included, all admissions were required to have undergone a radiography or a computed tomography scan of their chest on admission; the result of this imaging study was not considered. All admissions also must have received at least one dose of an IV antibiotic potentially used for pneumonia (i.e., ceftriaxone) within 24 hours of admission (Supplemental Table 1) and have received greater than three days of continuous antibiotic therapy. Furthermore, to be eligible for an IV-to-oral conversion, patient-admissions needed to be taking other oral medications on days 2–3 and not be in the intensive care unit during this time frame. To ensure patient-admissions did not have indications for antibiotics aside from CAP, we excluded those with secondary discharge diagnosis codes for other infections (i.e., cellulitis, urinary tract infection). We excluded patient-admissions on antibiotics for longer than 14 days to mitigate potential confounding from chronic suppressive oral antibiotics (i.e., chronic obstructive pulmonary disease exacerbation) or those with more complicated forms of pneumonia. We excluded patient-admissions transferred from an outside facility since we could not accurately assess initial antibiotics. We also excluded those transferred to another acute-care facility because, in these cases, the total duration of antibiotics could not be captured. We excluded patient-admissions who had a lung transplant or cystic fibrosis, as oral antibiotic options may be limited in this population. We excluded patient-admissions diagnosed with *Staphylococcus aureus* bacteremia, as these patients are often treated with IV antibiotics for prolonged periods of time. Lastly, we excluded those who died on or before day three.

### Variables

Demographics such as age at admission, sex, and race were collected as patient-level data. For each patient-admission, we also collected vital signs (temperature, heart rate, systolic blood pressure, and respiratory rate) from the period 24 hours before and 24 hours after the time of admission. Laboratory values (albumin, bilirubin, blood urea nitrogen, creatinine, glucose, sodium, hematocrit, white blood cell count) were gathered within 24 hours before and 48 hours after the time of admission. The most extreme values were used in a modified Acute Physiology and Chronic Health Evaluation (APACHE) III score.^11^ Missing values were assumed to be normal. Any positive blood cultures (excluding contaminants) collected on the day before admission through the time of hospital discharge were captured.

We used a modified version of the Elixhauser comorbidity index to identify comorbidities, based on ICD-10 codes from outpatient and inpatient encounters over the 12 months prior to the index visit and from the index admission itself.^12^

Diagnoses of bacterial infection (e.g., pneumonia) were based on ICD-10 codes applied at hospital discharge, as defined by a publicly available Agency for Healthcare Research and Quality resource.^13^

Antibiotics administered during the hospital stay were identified using the Barcode Medication Administration pharmacy data domain of the CDW. For each patient-admission, we collected data on all antibacterials (hereafter “antibiotics”) included in the National Healthcare Safety Network’s Antimicrobial Use and Resistance Protocol. Antibiotics specific to treating pneumonia were also identified (Supplemental Table 1).^14^ Using outpatient medication files, we identified postdischarge antibiotics; these were oral antibiotics dispensed from the outpatient pharmacy during the discharge period.^15^ We assumed that all outpatient oral antibiotics dispensed during this time frame were initiated by the patient on the day following discharge.

Missing data was rare except for laboratory values of albumin and bilirubin, which were either not checked or were missing in 9.0% and 12.8% of patient-admissions, respectively.

### Outcomes

The primary outcome was an early switch from IV-to-oral antibiotics within 72 hours of hospital admission (i.e., by three days of therapy). Both patient-admissions who were discharged before 72 hours and those who were still hospitalized at 72 hours were eligible for this outcome. We defined switching as discontinuation of all IV antibiotics and initiation of oral antibiotics without delay in therapy (based on conventional dosing intervals).^8^ We chose to evaluate early switch practices by day three of therapy since most patients admitted with community acquired pneumonia can be switched from IV-to-oral antibiotics after two to four days of therapy.^10^

Our secondary outcome was a composite measure of mortality and/or hospital readmission to a VHA hospital within 30 days of hospital discharge. Mortality and readmission can be competing risks, which is why we measured them as an either/or outcome. We obtained dates of death from the VHA Vital Status File, which combines mortality data from multiple VHA and non-VHA sources; this file strongly aligns with the National Death Index.^16^

### Statistical analysis

Descriptive statistics were summarized using medians, interquartile ranges, means, and standard deviations.

Among patient-admissions still hospitalized on day 3, we constructed a log-binomial regression model to identify factors associated with an early switch. We included a random intercept for each hospital to account for clustering of patients within each facility.

To account for the possibility that hospitals with lower switch rates had a different patient case-mix, we calculated an expected switch rate for each hospital. These rates were determined by first calculating the likelihood of an IV-to-oral switch for each patient-admission, using patient-level variables in a log-binomial regression model (independent of hospital). Patient-level variables included in the model were age, sex, BMI, 13 comorbidities, hospitalization in the past 90 days (yes/no), a modified APACHE score on admission, the year of hospitalization, and the presence of bacteremia. Next, we calculated the expected switch rate for a hospital as the mean expected value across its patients. Calculating an expected value for each hospital in this manner allowed us to account for inter-hospital differences in patient case-mix. The observed (or actual) frequency of switching was divided by the expected frequency to calculate an observed-to-expected (O:E) ratio for each hospital.^17^ Hospitals were grouped into quartiles based on their O:E ratios and the secondary outcome (death and/or readmission at 30 days) was compared across quartiles using a Kruskal Wallis test. All statistical tests were conducted using SAS version 9.4 and were two-tailed with *α* = 0.05.

## Results

### Analysis of patient-admissions

**Figure 1.**
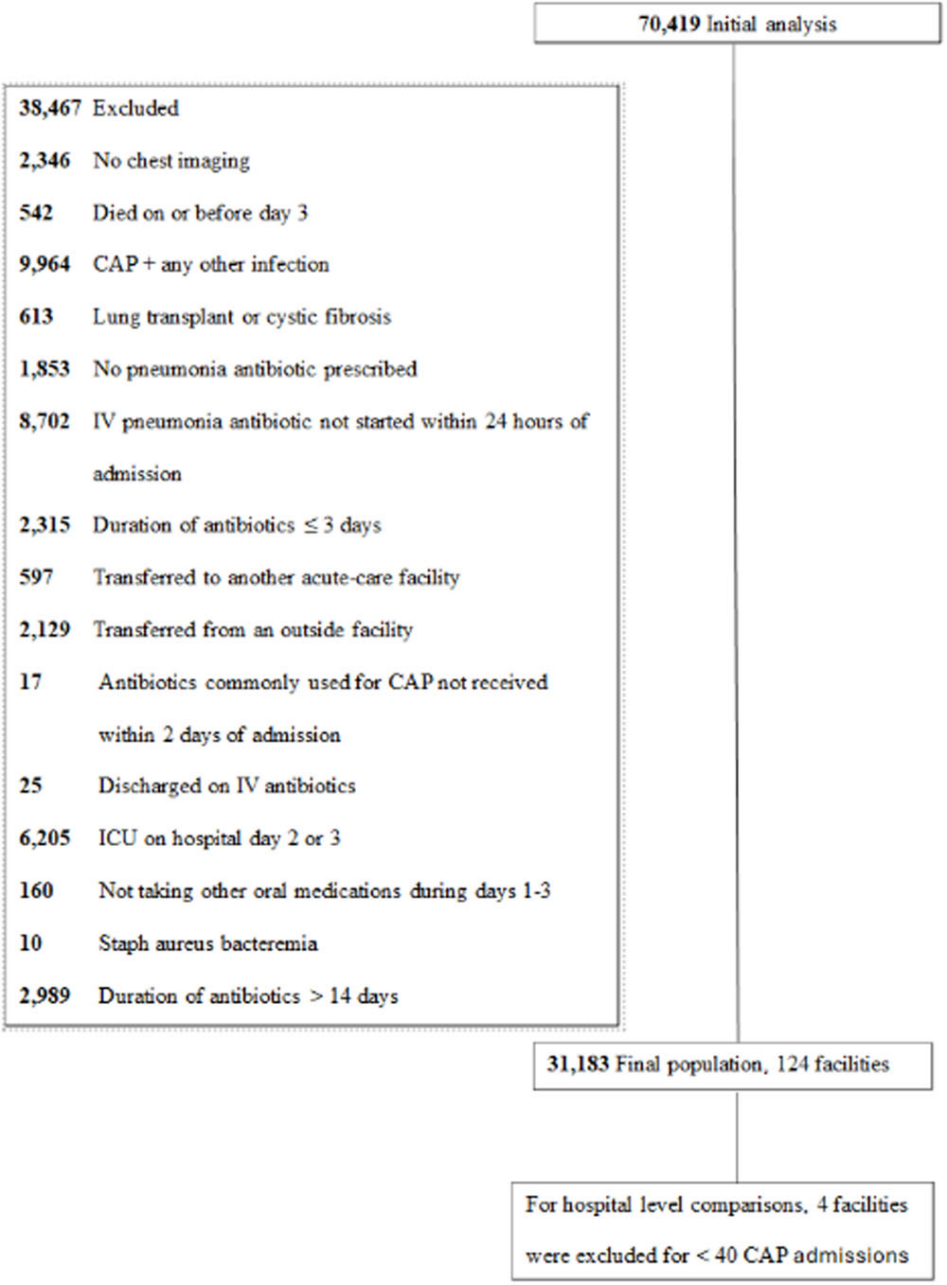
Patient selection flowchart for patients hospitalized with community-acquired pneumonia in the Veterans Health Administration, 2018–2023.

After applying the exclusion criteria, the final population consisted of 31,183 patient-admissions across 124 hospitals (Figure 1). Table 1 shows the clinical and demographic characteristics of those switched from IV-to-oral antibiotics versus those not switched. The median age was 73 years, and 29,805 (95.6%) participants were male. 21,703 (71.9%) patient-admissions were started on standard empiric CAP regimens (12,600 anti-pneumococcal beta-lactam monotherapy, 7,791 anti-pneumococcal beta-lactam + atypical therapy, 1,312 fluoroquinolone monotherapy) while 8,498 (28.1%) patients were started on broad-spectrum antibiotics (broad-spectrum gram-negative rod coverage and/or methicillin resistant staph aureus coverage).

**Table 1.** Clinical characteristics of hospitalized patients with community-acquired pneumonia who switched and did not switch from IV to oral antibiotics within 72 hours

Factor	Total (n = 31,183)	Discharged on or before day 3 on oral antibiotics (n = 15,113)	Hospitalized on day 3, switched to oral (n = 2,169)	Hospitalized on day 3, not switched to oral (n = 13,901)
**Age, median (IQR)**	73 (67–80)	73 (66–79)	74 (68–83)	74 (68–81)
**Male gender, n (%)**	29,805 (95.6)	14,414 (95.4)	2,063 (95.1)	13,328 (95.9)
**Race, n (%)** White Black Other	24,215 (77.7) 5,232 (16.8) 1736 (5.6)	11,761 (77.8) 2,491 (16.5) 861 (5.7)	1,698 (78.3) 345 (15.9) 126 (5.8)	10,756 (77.4) 2,396 (17.2) 749 (5.4)
**Comorbidities, n (%)** Alcohol use disorder Chemotherapy CHF COPD Diabetes Dementia Dialysis Immunocompromised Liver disease Neurologic condition	4,371 (14.0) 956 (3.1) 11,778 (37.8) 20,876 (67.0) 13,942 (44.7) 3,679 (11.8) 971 (3.1) 2,130 (6.8) 4,208 (13.5) 4,243 (13.6)	1,966 (13.0) 406 (2.7) 4,772 (31.6) 9,542 (63.1) 6,642 (44.0) 1,256 (8.3) 373 (2.5) 950 (6.3) 1,828 (12.1) 1,588 (10.5)	347 (16.0) 72 (3.3) 959 (44.2) 1,497 (69.0) 923 (42.6) 359 (16.6) 79 (3.6) 139 (6.4) 306 (14.1) 387 (17.9)	2,058 (14.8) 478 (3.4) 6,047 (43.5) 9,837 (70.8) 6,377 (45.9) 2,064 (14.9) 519 (3.7) 1,041 (7.5) 2,074 (14.9) 2,268 (16.3)
**Hospital stay in past** **90 days**, **n (%)**	6,291 (20.2)	2,068 (13.7)	510 (23.5)	3,713 (26.7)
**Modified APACHE score on admit, median (IQR)**	39 (31–47)	37 (29–45)	41 (33–49)	41 (33–49)
**Initial empiric therapy** Beta-lactam (BL) alone^a^ BL + atypical coverage^b^ FQ alone Broad-spectrum^c^ Other	12,600 (40.4) 7,791 (25.0) 1,312 (4.2) 8,498 (27.3) 982 (3.2)	7,102 (47.0) 3,997 (26.5) 735 (4.9) 2,801 (18.5) 478 (3.2)	936 (43.2) 440 (20.3) 131 (6.0) 569 (26.2) 93 (4.3)	4,562 (32.8) 3,354 (24.1) 446 (3.2) 5,128 (36.9) 411 (3.0)
**Concomitant bacteremia, n (%)**	156 (0.5)	50 (0.3)	11 (0.5)	95 (0.7)
**Length of stay in days, median (IQR)**	3 (2–5)	2 (2–3)	4 (4–6)	5 (4–7)
**Clinical outcomes** Readmission Mortality Composite^d^	4,385 (14.1) 1,594 (5.1) 5,629 (18.1)	1,845 (12.2) 292 (1.9) 2,027 (13.4)	335 (15.4) 122 (5.6) 425 (19.6)	2,205 (15.9) 1,180 (8.5) 3,177 (22.9)

Note. APACHE, acute physiology and chronic health evaluation; BL, beta-lactam antibiotic; CHF, congestive heart failure; COPD, chronic obstructive pulmonary disease; FQ, fluoroquinolone; IQR, interquartile range; IV, intravenous; SUD, substance use disorder.

a Beta-lactam empiric antibiotics were defined as receipt of any of the following agents within 24 hours of admission: ampicillin-sulbactam, cefotaxime, or ceftriaxone.

b Empiric antibiotics with atypical coverage were defined as receipt of any of the following agents within 24 hours of admission: azithromycin, clarithromycin, doxycycline, tetracycline, or minocycline.

c Broad-spectrum empiric antibiotics were defined as receipt of any of the following agents within 24 hours of admission: aztreonam, cefepime, ceftaroline, ceftazidime, ertapenem, imipenem-cilastatin, linezolid, meropenem, piperacillin-tazobactam, tedizolid, or vancomycin.

d The composite outcome indicates that within 30 days of the admission, the patient was either re-hospitalized and/or died.

**Figure 2.**
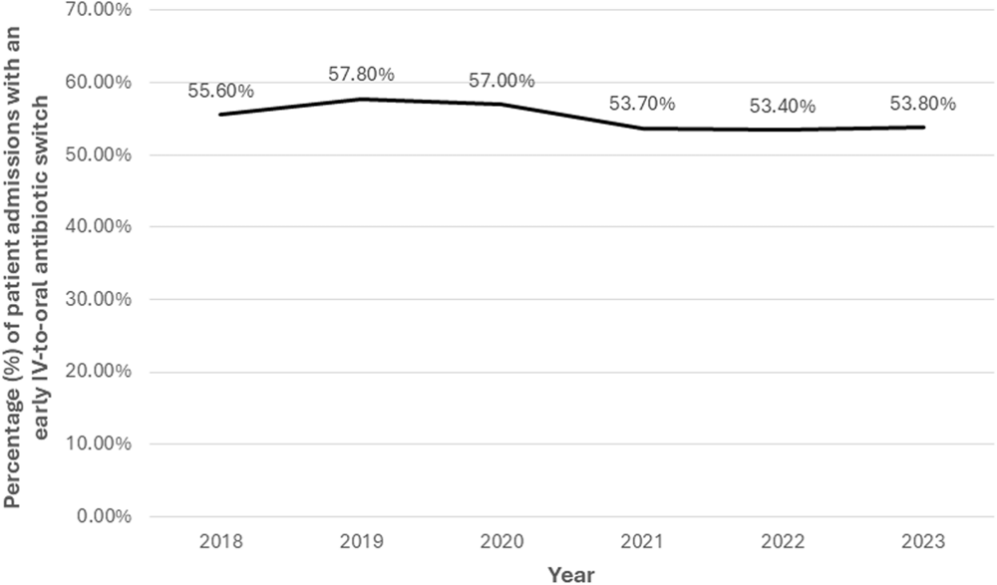
Frequency of early IV-to-oral antibiotic switches among all patients hospitalized with community-acquired pneumonia in the Veterans Health Administration, 2018–2023. “Early IV-to-oral antibiotic switch” is defined as discontinuation of all IV antibiotics and initiation of exclusively oral antibiotics without delay in therapy (based on conventional dosing intervals) within 72 hours of admission.

Overall, 17,282 (55.4%) patient-admissions underwent an early switch from IV-to-oral antibiotics. There was no clinically meaningful change in the proportion switched from IV-to-oral antibiotics over time in the entire cohort (Figure 2). However, patient-admissions hospitalized during 2019 were more likely to be switched than those hospitalized in 2018, after adjusting for other factors associated with switching (OR 1.05, 95% CI 1.02–1.08; Supplemental Table 2).

Among all 17,282 patient-admissions who switched early, 15,113 (87.4%) were switched to oral antibiotics and discharged by day three, including 3,270 (21.6%) who switched prior to discharge and 11,843 (78.4%) who switched at the time of discharge. The other 2,169 (12.6%) were switched to oral antibiotics while still in the hospital on day three. For those who had an early switch and were discharged by day three (*n* = 15,113), the most common oral regimens at discharge were a beta-lactam plus atypical coverage (40.1%), beta-lactam monotherapy (28.2%), or fluoroquinolone monotherapy (19.0%)

**Figure 3.**
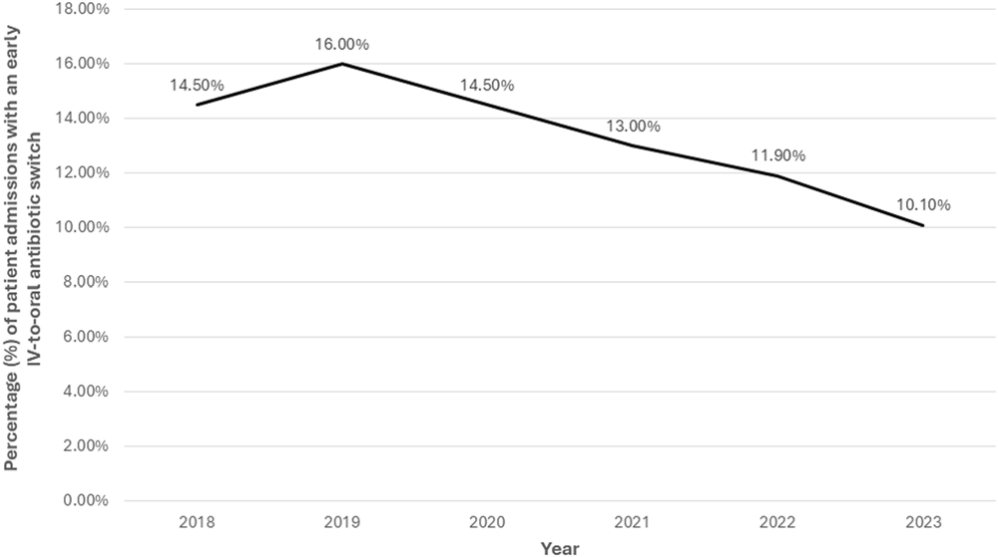
Frequency of early IV-to-oral antibiotic switches among patients with community-acquired pneumonia who were hospitalized through day three in the Veterans Health Administration, 2018–2023. “Early IV-to-oral antibiotic switch” is defined as discontinuation of all IV antibiotics and initiation of exclusively oral antibiotics without delay in therapy (based on conventional dosing intervals) within 72 hours of admission.

Of the 16,070 patient-admissions hospitalized through day three, 2,169 (13.5%) were switched to oral antibiotics. Figure 3 shows how the frequency of switching changed over time among these patients. The most common oral regimens in these admissions were a beta-lactam plus atypical coverage (31.8%), atypical monotherapy (27.6%), fluoroquinolone monotherapy (15.2%), and beta-lactam monotherapy (13.4%). Patient-level factors associated with an early switch among those still hospitalized on day three were older age (OR 1.009; 95% CI 1.004–1.013, *P* < .001) and having had received empiric fluoroquinolone monotherapy (OR 1.619; 95% CI 1.378–1.902, *P* < .001). Factors associated with not switching for those hospitalized through day three were having a more recent admission (2022 OR 0.779, 95% CI 0.681–0.891; 2023 OR 0.668; 95% CI 0.582–0.767), empiric therapy with a beta-lactam plus atypical coverage (OR 0.808; 95% CI 0.725–0.900), and empiric therapy with broad-spectrum antibiotics (OR 0.657; 95% CI 0.594–0.727) (Supplemental Table 3).

There were 4,385 (14.06%) patient-admissions who were re-admitted within 30 days and 1,594 (5.11%) died within 30 days. Overall, 5,979 (19.2%) were either re-admitted or died within this time frame.

### Hospital-level analyses

**Figure 4.**
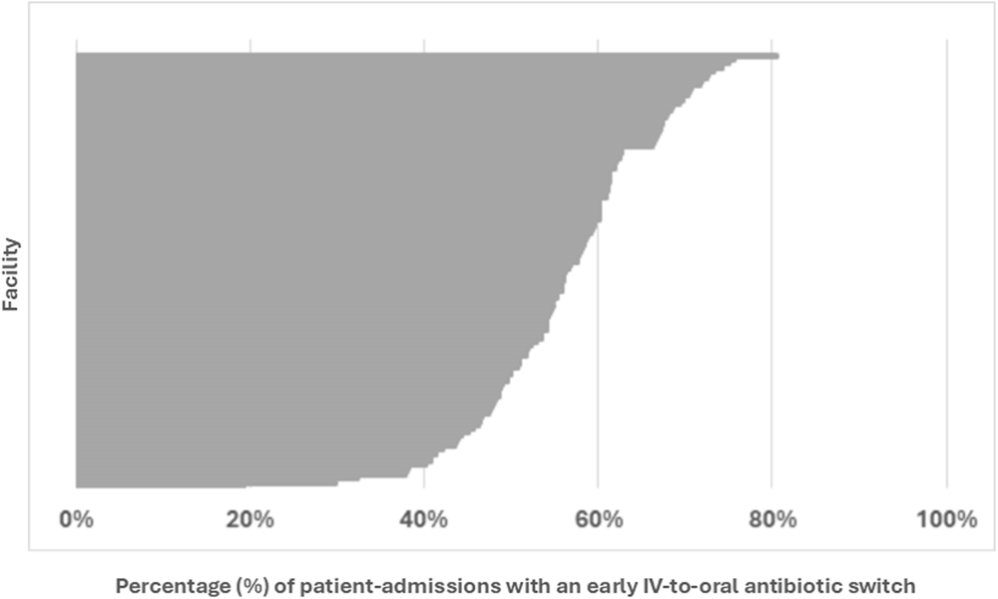
Frequency at which each hospital performed early IV-to-oral antibiotic conversions in qualifying patients with community acquired pneumonia, 2018–2023. This caterpillar plot displays the 120 facilities included in the hospital-level analysis and the associated frequency at which each hospital performed an early IV-to-oral antibiotic switch. Numerators and denominators by year are as follows: 2018 (3588/6449), 2019 (3780/6536), 2020 (2568/4508), 2021 (2191/4077), 2022 (2363/4427), 2023 (2792/5186). “Early IV-to-oral antibiotic switch” is defined as discontinuation of all IV antibiotics and initiation of exclusively oral antibiotics without delay in therapy (based on conventional dosing intervals) within 72 hours of admission.

**Figure 5.**
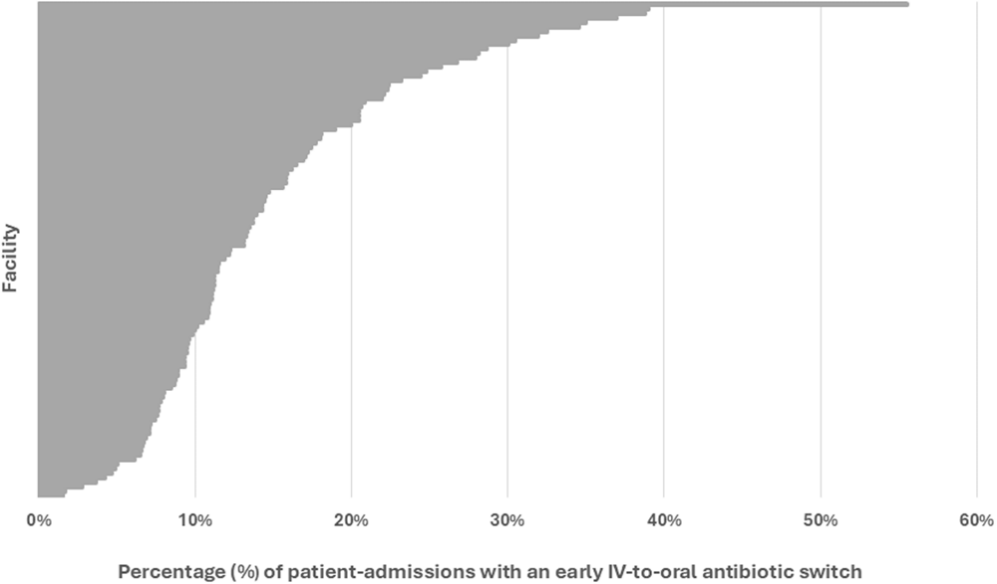
Frequency at which each hospital performed early IV-to-oral antibiotic conversions in qualifying patients with community acquired pneumonia who were hospitalized through day three, 2018–2023. This caterpillar plot displays the 111 facilities included in the hospital-level analysis and the associated frequency at which each hospital performed an early IV-to-oral antibiotic switch. Numerators and denominators by year are as follows: 2018 (487/3348), 2019 (524/3280), 2020 (328/2268), 2021 (282/2168), 2022 (279/2343), 2023 (269/2663). “Early IV-to-oral antibiotic switch” is defined as discontinuation of all IV antibiotics and initiation of exclusively oral antibiotics without delay in therapy (based on conventional dosing intervals) within 72 hours of admission.

Four hospitals were excluded from the analysis across all patients (i.e., both early discharges and patients still hospitalized on day three) since these hospitals had fewer than 40 admissions for CAP. The median hospital rate of early switching from IV-to-oral antibiotics across all patients was 56.8% (IQR 49.6%–62.2%) (Figure 4). An additional nine hospitals were excluded from the analysis of patients still hospitalized through day three. The median hospital rate of early switching from IV-to-oral antibiotics in these patients was 12% (IQR 9–19%) (Figure 5).

Hospitals were ranked into quartiles based on their O:E ratio for early IV-to-oral antibiotic switches. Hospitals with lower O:E ratios were grouped into quartiles one and two while those with higher O:E ratios were in quartiles three and four. Expected IV-to-oral antibiotic switch rates were similar across all hospitals while the O:E ratio for switches ranged from 0.78 among hospitals in quartile one to 1.23 in quartile four. Supplemental Table 4 shows the characteristics of the hospitals included in this analysis grouped by quartile. Rates of switching from IV-to-oral antibiotics did not differ by hospital complexity level across quartiles (Kruskal-Wallis *χ* ^2^ = 7.3, *P* = .12). However, in a posthoc analysis, level 1a hospitals demonstrated a higher rate of switching to oral antibiotics compared to all other complexity levels (*P* = .01). There was no difference in the composite outcome of mortality and/or readmission across quartiles (Kruskal-Wallis *χ* ^2^ = 7.4; *P* = .06) (Table 2).

**Table 2.** Hospital-level observed-to-expected (O:E) frequencies of early IV-to-oral antibiotic switches for community-acquired pneumonia and associated clinical outcomes, stratified by O:E quartiles for 120 hospitals

Parameter	Quartile 1 median (IQR)	Quartile 2 median (IQR)	Quartile 3 median (IQR)	Quartile 4 median (IQR)
**Hospitalizations, n**	228 (154, 360)	244 (172, 396)	247 (153, 331)	220 (151, 271)
**IV-to-oral switches**				
Observed, %	44.1 (40.2, 48.8)	54.1 (50.9, 55.3)	60.1 (58.4, 61.3)	68.5 (66.8, 71.9)
Expected, %	56.4 (55.4, 57.4)	55.4 (54.0, 56.0)	55.5 (54.4, 56.8)	55.7 (54.1, 57.3)
O:E ratio	0.78 (0.72, 0.87)	0.98 (0.93, 1.00)	1.07 (1.05, 1.09)	1.23 (1.20, 1.27)
**Outcomes**				
30-day mortality, %	4.6 (3.4, 6.5)	4.9 (4.2, 6.4)	4.5 (3.2, 5.5)	4.2 (3.5, 5.5)
Readmission, %	12.1 (8.5, 14.0)	14.8 (13.2, 16.4)	13.5 (11.5, 16.5)	13.6 (10.7, 15,9)
Composite, %	15.7 (12.6, 17.7)	18.7 (17.1, 20.0)	17.5 (15.1, 20.5)	16.8 (14.4, 19.9)

## Discussion

In this nationwide cohort of patients admitted to VA hospitals with CAP, slightly more than half were transitioned to oral antibiotics on or before day three of therapy. Among patient-admissions with an early switch to oral antibiotics, two out of three were switched at the time of discharge. The frequency of IV-to-oral antibiotic switches varied widely across hospitals but has been stable over time. In line with prior studies, we found that outcomes among patients at hospitals with high switch rates were comparable to outcomes at hospitals with low switch rates, thereby supporting the safety of early conversions.^8^

Despite evidence supporting early switch (by day three of therapy) from IV-to-oral antibiotics for patients hospitalized with CAP, uptake of this practice remains low. A nationwide cohort study published by Deshpande et. al. in 2023 found that 6% of patients hospitalized with CAP were switched to oral antibiotics by day three of hospitalization, though early switch was associated with reduced length of stay and hospitalization cost without an increase in mortality rates.^8^ Compared to the Deshpande et. al. study, our study found that among patients who remained in the hospital on day three of therapy, 13% were switched from IV-to-oral antibiotics, which is only marginally greater than 6%. In line with Deshpande et. al. study, our study found that early switch was not associated with worse composite clinical outcomes. However, in contrast to Deshpande et al, we included patients who were discharged on oral antibiotics before day three, a group that represented nearly half of hospitalized patients with CAP and seven out of every eight patients who had an early conversion.

Our finding that the percentage of expected IV-to-oral switches was consistent across quartiles may suggest that local practice patterns are driving variations in IV-to-oral switch rates more than differences in patient case-mix. A less likely, albeit possible, explanation is that there were key patient-level factors associated with switching or not switching that we did include in our model. If differences in local practice patterns play a large role in whether patients switch, this would be consistent with prior reports on how social norms influence antibiotic-prescribing.^18,19^

Early IV-to-oral antibiotic switches in patients hospitalized with CAP remains an unrealized goal of antimicrobial stewardship programs. This may, in part, be due to choice of initial antibiotics in this population. Our study found that the use of broad-spectrum IV antibiotics was common initially, which may have served as a barrier to de-escalate therapy to oral antibiotics. Another potential reason for poor implementation of IV-to-oral antibiotic protocols may be due to the following provider-level concerns: (1) the belief that IV antibiotics are superior to oral antibiotics, (2) hesitancy to switch if there is not a direct oral equivalent to the IV formulation, (3) time constraints, or (4) limited knowledge of the patient due to the frequent rotation of physicians.^20,21^ Pharmacist-initiated IV-to-oral antibiotic switches when defined criteria are met as well as clinical decision support tools for providers may be effective strategies to address the above-mentioned barriers and ensure timely IV-to-oral switches.^22–24^ These switches can shorten length of stay, reduce nursing workload, decrease costs, and have environmental benefits.^25^

Opportunities for early IV-to-oral antibiotic switches may change as clinical practice evolves to support shorter courses of antibiotic therapy for many infections, including CAP. Our study did not assess adherence to shorter durations of therapy for CAP, though this will likely be an area of interest for future studies.

### Strengths and limitations

Compared to previously published literature, our nationwide study included patients who were discharged before day three, which represented nearly half of the patients hospitalized for CAP. Another strength of our analysis is that we accounted for inter-facility differences in patient case-mix by calculating an O:E ratio for IV-to-oral switches at each hospital.

However, our study has limitations. First, we were unable to assess readmission rates if patients presented to a non-VA hospital. Second, we were unable to assess clinical status at the time of switching, nor did we exclude patients who failed to meet criteria for clinical stability by day three. While we may have overestimated the percentage of patients who could have switched, prior work has indicated that the median time to clinical stability in patients with CAP is three days.^26^ Third, we did not evaluate whether microbiologic findings would have permitted an IV-to-oral antibiotic switch, but because an antibiotic-resistant organism is rarely identified in CAP, we suspect the effect of this omission was likely minimal.^27^ Fourth, we were unable to confirm whether patients in this cohort actually had CAP, a condition that is frequently over-diagnosed.^28^ Fifth, we did not exclude patients who were referred to hospice and, in turn, may have requested less aggressive care (e.g., oral instead of IV antibiotics). Sixth, our study overlapped with the COVID-19 pandemic, and it is unclear how the pandemic influenced the outcome of our study. Finally, our findings may not be generalizable to non-VA hospitals given the predominately older white male population.

## Conclusion

In conclusion, our study found that early IV-to-oral antibiotic switch by day three of therapy for patients hospitalized with community acquired pneumonia appears safe but is underutilized. Overall, our study indicates a need to encourage stewardship programs and inpatient providers to pay greater attention to opportunities for switching to oral antibiotics among patients who qualify.

## Supporting information

Daniels et al. supplementary material Daniels et al. supplementary material

## Supplementary material

The supplementary material for this article can be found at https://doi.org/10.1017/ice.2025.10389
