# Supplementary material for: Frequency of intravenous-to-oral antibiotic switch in VA hospitalized patients with community-acquired pneumonia
Source: Infect Control Hosp Epidemiol. 2026 Feb 2;47(4):365–72. doi: 10.1017/ice.2025.10389 (PMC13216802; doi:10.1017/ice.2025.10389)
Supplement: Daniels et al. supplementary material [file S0899823X25103899sup001.docx]

**Supplemental Table 1. Antibiotics that could potentially be used to treat pneumonia, either empirically or based on microbiologic findings**

| **Antibiotic Name** | **Categorization for empiric antibiotic therapy** |
| --- | --- |
| Ampicillin-sulbactam | Beta-lactam for CAP |
| Azithromycin | Atypical coverage |
| Aztreonam | Broad-spectrum antibiotic for gram-negative coverage |
| Cefepime | Broad-spectrum antibiotic for gram-negative coverage |
| Cefotaxime | Beta-lactam for CAP |
| Ceftaroline | Broad-spectrum antibiotic for MRSA coverage |
| Ceftazidime | Broad-spectrum antibiotic for gram-negative coverage |
| Ceftriaxone | Beta-lactam for CAP |
| Clindamycin | Other antibiotic |
| Ciprofloxacin | Fluoroquinolone |
| Clarithromycin | Atypical coverage |
| Doxycycline | Atypical coverage |
| Ertapenem | Broad-spectrum antibiotic for gram-negative coverage |
| Imipenem-cilastatin | Broad-spectrum antibiotic gram-negative coverage |
| Levofloxacin | Fluoroquinolone |
| Linezolid | Broad-spectrum antibiotic for MRSA coverage |
| Meropenem | Broad-spectrum antibiotic for gram-negative coverage |
| Minocycline | Atypical coverage |
| Moxifloxacin | Fluoroquinolone |
| Piperacillin-tazobactam | Broad-spectrum antibiotic for gram-negative coverage |
| TMP/SMX (intravenous) | Other antibiotic |
| Tedizolid | Broad-spectrum antibiotics for MRSA coverage |
| Tetracycline | Atypical coverage |
| Vancomycin (intravenous) | Broad-spectrum antibiotic for MRSA coverage |

Abbreviations: CAP community acquired pneumonia; MRSA methicillin-resistant Staphylococcus aureus; TMP/SMX trimethoprim-sulfamethoxazole

**Supplemental Table 2. Relative risk of an early IV-to-oral antibiotic switch in patients hospitalized with community-acquired pneumonia, based on the log-binomial regression model used to estimate the expected likelihood of switching***

| **Variable** | **Odds Ratio (95% CI)** | **p-value** |
| --- | --- | --- |
| **Age (per year)** | 1.000 (0.999-1.001) | 0.806 |
| **Female sex** | 0.956 (0.916-0.998) | 0.042 |
| **BMI**  Missing  Underweight  Overweight  Obese | 0.974 (0.878-1.081)  0.904 (0.859-0.952)  1.051 (1.025-1.077)  1.066 (1.039-1.093) | 0.622  <0.001  <0.001  <0.001 |
| **Comorbidities**  Alcohol SUD  Cancer  Chemotherapy  CHF  COPD  Diabetes mellitus Dialysis  Drug SUD  Immunocompromised  Liver disease  Neurologic condition  Paralysis  Rheumatic disease | 0.961 (0.933-0.990)  0.998 (0.975-1.022)  0.948 (0.990-1.009)  0.893 (0.873-0.913)  0.923 (0.906-0.941)  1.013 (0.993-1.034)  0.998 (0.934-1.067)  0.973 (0.943-1.003)  0.980 (0.940-1.023)  0.957 (0.928-0.987)  0.871 (0.842-0.900)  0.933 (0.860-1.013)  0.964 (0.920-1.011) | 0.009  0.850  0.094  <0.001  <0.001  0.196  0.958  0.080  0.358  0.005 <0.001  0.100  0.133 |
| **Hospital stay in the past 90 days** | 0.758 (0.734-0.782) | <0.001 |
| **Modified APACHE score on admission (per point)** | 0.991 (0.990-0.992) | <0.001 |
| **Year (reference 2018)**  **2019**  **2020**  **2021**  **2022**  **2023** | 1.047 (1.018-1.076)  1.029 (0.998-1.060)  0.989 (0.956-1.023)  0.980 (0.949-1.013)  0.995 (0.964-1.026) | 0.001  0.065  0.514  0.239  0.741 |
| **Bacteremia** | 0.775 (0.640-0.939) | 0.009 |

Abbreviations: APACHE acute physiology and chronic health evaluation; BMI body mass index; CHF congestive heart failure; COPD chronic obstructive pulmonary disease; SUD substance use disorder

*Empiric antibiotic therapy was not included in this model because what was prescribed empirically may not have been guideline-concordant.

**Supplemental Table 3. Relative risk of an early IV-to-oral antibiotic switch among patients with community-acquired pneumonia who were hospitalized through day 3 (n=16,070)**

| **Variable** | **Relative risk (95% CI)** | **p-value** |
| --- | --- | --- |
| **Age (per year)** | 1.009 (1.004-1.013) | <0.001 |
| **Female sex** | 1.167 (0.984-1.385) | 0.075 |
| **BMI**  Missing  Underweight  Overweight  Obese | 0.843 (0.522-1.362)  0.975 (0.835-1.138)  0.920 (0.836-1.013)  0.925 (0.836-1.023) | 0.485  0.745  0.091  0.129 |
| **Comorbidities**  Alcohol SUD  Cancer  Chemotherapy  CHF  COPD  Diabetes mellitus Dialysis  Drug SUD  Immunocompromised  Liver disease  Neurologic condition  Paralysis  Rheumatic disease | 1.081 (0.967-1.209)  0.895 (0.816-0.981)  1.066 (0.859-1.324)  1.046 (0.965-1.135)  0.940 (0.865-1.021)  0.958 (0.883-1.040)  1.053 (0.854-1.298)  1.002 (0.889-1.129)  0.979 (0.831-1.154)  1.003 (0.894-1.125)  1.092 (0.988-1.206)  1.087 (0.849-1.392)  0.871 (0.714-1.063) | 0.172  0.017  0.562  0.277  0.142  0.307  0.632  0.980  0.803  0.960  0.084  0.508  0.176 |
| **Hospital stay in the past 90 days** | 0.943 (0.857-1.036) | 0.221 |
| **Modified APACHE score on admission (per point)** | 0.996 (0.992-0.999) | 0.018 |
| **Year** (reference 2018)  **2019**  **2020**  **2021**  **2022**  **2023** | 1.052 (0.944-1.173)  0.983 (0.868-1.112)  0.871 (0.762-0.995)  0.779 (0.681-0.891)  0.668 (0.582-0.767) | 0.358  0.782  0.042  <0.001  <0.001 |
| **Empiric antibiotic therapy**  (reference beta-lactam monotherapy)  Beta-lactam combination therapy  Fluoroquinolone monotherapy  Broad-spectrum antibiotics  Other antibiotics | 0.808 (0.725-0.900)  1.619 (1.378-1.902)  0.657 (0.594-0.727)  1.149 (0.957-1.381) | <0.001  <0.001  <0.001  0.136 |
| **Bacteremia** | 0.814 (0.472-1.405) | 0.460 |

Abbreviations: APACHE acute physiology and chronic health evaluation; BMI body mass index; CHF congestive heart failure; COPD chronic obstructive pulmonary disease; SUD substance use disorder

**Supplemental Table 4. Hospital characteristics and frequencies of early IV-to-oral antibiotic switches, stratified by observed-to-expected (O:E) ratio quartiles for 120 hospitals**

| **Hospital Characteristics** | **Quartile 1, no. (%) (n = 30)** | **Quartile 2, no. (%) (n = 30)** | **Quartile 3, no. (%) (n = 30)** | **Quartile 4, no. (%) (n = 30)** |
| --- | --- | --- | --- | --- |
| **Hospital Complexity**  Level 1a  Levels 1b-1c  Levels 2-3 | 5 (16.7%)  13 (43.3%)  12 (40.0%) | 12 (40%)  12 (40%)  6 (20%) | 9 (30%)  9 (30%)  12 (40%) | 16 (53.3%)  8 (26.7%)  6 (20%) |
| **Urban** | 26 (86.7%) | 29 (96.7%) | 25 (83.3%) | 28 (93.3%) |
| **Bed Size**  ≤ 50 beds  51-99 beds  ≥ 100 beds | 20 (66.7%)  2 (6.8%)  8 (26.7%) | 6 (20%)  14 (46.7%)  10 (33.3%) | 9 (30%)  12 (40%)  9 (30%) | 9 (30%)  8 (26.7%)  13 (43.3%) |
| **Region**  Midwest  Northeast  South  West | 7 (23.3%)  4 (13.3%)  16 (53.3%)  3 (10.0%) | 4 (13.3%)  6 (20%)  15 (50%)  5 (16.7%) | 11 (36.7%)  5 (16.7%)  8 (26.7%)  6 (20%) | 7 (23.3%)  6 (20%)  9 (30%)  8 (26.7%) |

Hospitals were grouped into quartiles based on their observed-to-expected (O:E) ratio for early IV-to-oral antibiotic switches. Median O:E ratios ranged from 0.78 for hospitals in quartile one to 1.23 for hospitals in quartile four. VA hospitals are scored according to their patient population, clinical services (e.g. intensive care unit [ICU] and surgery services), and education and research. A score of 1a is the most complex while a score of 3 is the least complex.
